# Prognostic nutritional index and geriatric nutritional risk index as predictors of in-hospital major adverse cardiovascular events after percutaneous coronary intervention in acute coronary syndrome patients: a multicenter retrospective cohort study

**DOI:** 10.3389/fnut.2026.1848510

**Published:** 2026-08-26

**Authors:** Jun Li, Fachao Shi, Zheng Wang, Caoyang Fang

**Affiliations:** 1 Department of Cardiology, Chongming Hospital Affiliated to Shanghai University of Medicine and Health Sciences, Shanghai, China; 2 Department of Cardiology, Maanshan People's Hospital, Maanshan, China; 3 Department of Cardiology, The Second People's Hospital of Hefei, Hefei Hospital Affiliated to Anhui Medical University, Hefei, China; 4 Department of Emergency, First Affiliated Hospital of University of Science and Technology of China, Anhui Provincial Hospital, Hefei, China

**Keywords:** acute coronary syndrome, geriatric nutritional risk index, major adverse cardiovascular events, multicenter study, percutaneous coronary intervention, prognostic nutritional index

## Abstract

**Objective:**

To evaluate the association between PNI and GNRI with in-hospital MACE after PCI in patients with ACS, and to compare the predictive value of these two nutritional assessment tools. The optimal cutoff values of PNI and GNRI were determined by calculating the maximum Youden’s index based on ROC curve analysis in the training cohort.

**Methods:**

This multicenter retrospective cohort study included ACS patients who underwent PCI at three hospitals between January 2021 and December 2024. The training cohort consisted of 638 patients from Anhui Provincial Hospital, while the validation cohort included 212 patients from Hefei Second People’s Hospital and 238 patients from Ma’anshan People’s Hospital. PNI was calculated based on serum albumin and lymphocyte count at admission, while GNRI was calculated using serum albumin and body mass index. The primary endpoint was in-hospital MACE, including all-cause mortality, myocardial infarction, stroke, and target vessel revascularization. Multivariate logistic regression analysis was used to assess the association of PNI and GNRI with in-hospital MACE, and the predictive abilities of the two indices were compared using ROC curve analysis.

**Results:**

In the training cohort, 63 patients (9.9%) experienced in-hospital MACE. Multivariate analysis showed that low PNI (<45.2) and low GNRI (<98.0) were both independent predictors of in-hospital MACE (PNI: adjusted OR = 2.37, 95%CI: 1.42–3.96, *p* = 0.001; GNRI: adjusted OR = 2.18, 95%CI: 1.31–3.64, *p* = 0.003). ROC analysis revealed that PNI (AUC = 0.721, 95%CI: 0.653–0.789) and GNRI (AUC = 0.703, 95%CI: 0.632–0.774) had similar predictive values (*p* = 0.247). Similar results were observed in the validation cohort, and DeLong test confirmed no significant difference in AUC values of the two indices between training and validation cohorts, confirming the reliability of these findings. The combination of both nutritional indices (AUC = 0.753, 95%CI: 0.687–0.819) showed superior predictive ability compared to either index alone.

**Conclusion:**

Both PNI and GNRI serve as effective predictive tools for in-hospital MACE after PCI in ACS patients. The two nutritional assessment tools have similar predictive capabilities, and their combination may provide more accurate risk assessment. Routine nutritional status evaluation in ACS patients helps identify high-risk individuals, providing a basis for early intervention.

## 1 Introduction

Acute Coronary Syndrome (ACS) is a leading cause of cardiovascular mortality and disability worldwide. Despite significant improvements in patient prognosis through Percutaneous Coronary Intervention (PCI), post-procedural Major Adverse Cardiovascular Events (MACE) remain a focal concern in clinical practice (1). Globally, approximately 7.6 million people die from coronary heart disease annually, while both the incidence and mortality rates of ACS in China show an upward trend, posing a serious threat to public health (2, 3). In recent years, numerous studies have demonstrated that nutritional status is closely associated with cardiovascular disease outcomes. Malnutrition can influence disease prognosis through various mechanisms, including promoting vascular inflammatory responses, accelerating atherosclerosis progression, and compromising immune function (4). However, traditional nutritional assessment methods are complex and have limited clinical application. Therefore, simple and reliable nutritional assessment tools have significant value in optimizing the management of ACS patients (5).

The Prognostic Nutritional Index (PNI) is a comprehensive scoring system based on serum albumin and lymphocyte count, initially used to assess the risk of postoperative complications in gastrointestinal surgery patients (6). The Geriatric Nutritional Risk Index (GNRI), which combines serum albumin and the ratio of actual to ideal body weight, was originally designed to evaluate nutritional risk in elderly patients (7). Both indices are easily calculated, requiring parameters readily available from routine clinical examinations, and have demonstrated good prognostic value in various diseases (8). Recent studies indicate that PNI effectively predicts adverse outcomes in patients with acute myocardial infarction, acute heart failure, and cardiac surgery, while GNRI has shown potential in predicting in-hospital mortality and long-term prognosis in patients with chronic heart failure (9, 10).

In the field of ACS, multiple studies have confirmed the correlation between PNI and both short-term and long-term patient prognosis. Cheng et al. found that low PNI values were significantly associated with in-hospital mortality in patients with ST-segment elevation myocardial infarction and provided incremental predictive value (9). Similarly, Keskin et al. reported that low PNI is an independent predictor of long-term all-cause mortality in patients with acute myocardial infarction (11). Shibata et al. confirmed that GNRI is an independent risk factor for early mortality after cardiac surgery (12). Nevertheless, research on the prognostic value of PNI and GNRI in ACS patients undergoing PCI is relatively limited, particularly regarding their combined application and evidence from Chinese multicenter ACS populations (13).

Notably, although both PNI and GNRI include serum albumin as a key indicator, they emphasize different aspects: PNI focuses more on immune status (through lymphocyte count), while GNRI concentrates on overall nutritional status (through weight indicators) (14). Research suggests that in certain disease states, these two indices may provide complementary prognostic information. Lee et al. found in their study of cirrhotic patients that the combined application of PNI and GNRI could improve the accuracy of prognosis prediction (15). However, the value of this combined assessment approach in ACS patients has not been thoroughly explored.

There exists a complex interaction between inflammatory response and nutritional status, which plays an important role in the pathophysiological process of ACS (16). Hypoalbuminemia not only reflects malnutrition but may also be a marker of systemic inflammatory status (17). Similarly, lymphocytopenia may reflect decreased immune function and persistent inflammation (18). Therefore, PNI and GNRI may predict the prognosis of ACS patients by assessing these interrelated pathological processes. Wu et al. (19) found that PNI was significantly associated with the severity and prognosis of coronary artery disease, while Bouillanne et al. confirmed that GNRI could predict adverse clinical outcomes and mortality in elderly hospitalized patients (7). Although previous studies have explored the relationship between PNI or GNRI and ACS prognosis, most were single-center studies with limited sample sizes and lacked systematic evaluation in Chinese ACS patients (8, 20). Furthermore, the optimal cutoff values for PNI and GNRI may differ across populations, which has not been adequately validated in Chinese ACS patients (21).

Based on the above background, this study aims to systematically evaluate the association between PNI and GNRI with in-hospital MACE in ACS patients undergoing PCI through a multicenter retrospective cohort design, and to explore the value of combining these two indices. The results are expected to provide clinicians with simple and practical risk stratification tools to optimize treatment decisions and prognosis management for ACS patients.

## 2 Methods

### 2.1 Study subjects

This study is a multicenter retrospective cohort study that included ACS patients who underwent PCI treatment between January 2021 and December 2024 at Anhui Provincial Hospital, Hefei Second People’s Hospital, and Ma’anshan People’s Hospital. The training cohort consisted of 638 patients from Anhui Provincial Hospital, while the validation cohort included 212 patients from Hefei Second People’s Hospital and 238 patients from Ma’anshan People’s Hospital.

Sample size justification and statistical power analysis was performed prior to cohort enrollment. Based on previously reported OR values of nutritional indices for cardiovascular adverse events and an expected in-hospital MACE rate of 10% in ACS patients, a minimum sample size of 526 was required to achieve a statistical power of 0.80 and a two-sided *α* of 0.05. The enrolled total sample size (*n* = 1,088) adequately met the statistical requirement and ensured sufficient statistical efficacy for subsequent regression analysis, subgroup comparison, and predictive model verification.

Inclusion criteria were: (1) age ≥18 years; (2) ACS diagnosis based on the Fourth Universal Definition of Myocardial Infarction (22) and the latest European Society of Cardiology guidelines (23), including ST-segment Elevation Myocardial Infarction (STEMI), Non-ST-segment Elevation Myocardial Infarction (NSTEMI), and Unstable Angina (UA); (3) receiving PCI treatment after admission. Exclusion criteria were: (1) severe infection, tumor, autoimmune disease, hematological disease, or severe liver or kidney dysfunction; (2) specific diseases and conditions that may significantly affect serum albumin and peripheral lymphocyte levels, including nephrotic syndrome, hypersensitivity reactions, hematologic malignancies, systemic lupus erythematosus, and other immune-mediated disorders; (3) history of surgery, trauma, or blood transfusion (≤3 months); (4) incomplete clinical data. During patient screening, a total of 57 patients were excluded due to incomplete key clinical data, mainly including missing admission lymphocyte count, serum albumin, height and weight, LVEF, NT-proBNP and CRP data, which were indispensable for nutritional index calculation and multivariate adjustment analysis. The study protocol was approved by the Ethics Committee of Anhui Provincial Hospital and adhered to the principles of the Declaration of Helsinki. Informed consent was waived due to the retrospective design of the study.

### 2.2 Data collection

Baseline clinical data were collected through the electronic medical record system, including: (1) Demographic characteristics: age, gender, weight, and height; (2) Underlying diseases: hypertension, diabetes, history of stroke, history of myocardial infarction, heart failure, chronic kidney disease, peripheral arterial disease, etc.; (3) Lifestyle factors: smoking and alcohol consumption; (4) Laboratory tests: white blood cell count, neutrophil count, lymphocyte count, hemoglobin, platelet count, creatinine, creatine kinase, troponin I, N-terminal pro-brain natriuretic peptide (NT-proBNP), total cholesterol, low-density lipoprotein cholesterol, high-density lipoprotein cholesterol, triglycerides, serum albumin, C-reactive protein(CRP); (5) Echocardiography: left ventricular ejection fraction (LVEF); (6) Coronary angiography and PCI-related information: number of diseased vessels, syntax score, number and types of stents, etc.; (7) Medication therapy: antiplatelet drugs, *β*-blockers, angiotensin-converting enzyme inhibitors/angiotensin II receptor blockers, statins, etc. All laboratory tests were based on the first results obtained upon admission.

### 2.3 Nutritional assessment index calculation

The Prognostic Nutritional Index (PNI) was calculated according to the following formula: PNI = 10 × serum albumin (g/dL) + 0.005 × total lymphocyte count (/mm^3^, 6). The Geriatric Nutritional Risk Index (GNRI) was calculated using the following formula: GNRI = 1.489 × serum albumin (g/L) + 41.7 × (actual weight/ideal weight) (7). Ideal weight was calculated using the Lorentz formula: for males = height (cm)-100-[(height(cm)-150)/4]; for females = height(cm)-100-[(height (cm)-150)/2.5]. If the actual weight/ideal weight ratio was greater than 1, it was set to 1. In the training cohort, the optimal cutoff values of PNI and GNRI for predicting in-hospital MACE were determined by ROC curve analysis with the maximum Youden’s index (sensitivity+specificity-1) as the screening criterion. Patients were divided into two groups according to PNI and GNRI: low PNI group (PNI < 45.2) and high PNI group (PNI ≥ 45.2); low GNRI group (GNRI < 98.0) and high GNRI group (GNRI ≥ 98.0).

### 2.4 Definition of endpoints

The primary endpoint of this study was in-hospital MACE, defined as the occurrence of any of the following events during hospitalization: all-cause mortality, recurrent myocardial infarction, stroke, or target vessel revascularization. Recurrent myocardial infarction was defined as the recurrence of chest pain after PCI, accompanied by re-elevation of myocardial necrosis markers (exceeding the 99th percentile of the upper limit of normal) or new ischemic changes on electrocardiogram. Stroke was defined as an acute cerebral ischemic or hemorrhagic event confirmed by imaging, with neurological deficits lasting ≥24 h. Target vessel revascularization was defined as repeat interventional treatment or surgical bypass grafting of the original target vessel after PCI.

### 2.5 Statistical analysis

Continuous variables were presented as mean ± standard deviation or median (interquartile range) according to their distribution characteristics, and between-group comparisons were performed using independent samples *t*-test or Mann–Whitney *U* test. Categorical variables were expressed as frequencies (percentages), and between-group comparisons were conducted using chi-square test or Fisher’s exact test.

Multivariable logistic regression analysis was used to evaluate the association between PNI, GNRI, and in-hospital MACE, with models adjusted for potential confounding factors including age, gender, body mass index (BMI), ACS type, Killip classification, hypertension, diabetes, previous myocardial infarction, chronic kidney disease, LVEF, three-vessel disease, syntax score, CRP, and NT-proBNP. Variance inflation factor (VIF) analysis was performed to assess multicollinearity among all included covariates. A VIF value <10 was defined as the threshold for the absence of significant multicollinearity. Results were presented as odds ratios (OR) with 95% confidence intervals (CI).

The predictive ability of PNI and GNRI for in-hospital MACE was assessed through receiver operating characteristic (ROC) curve analysis, calculating the area under the curve (AUC), sensitivity, specificity, positive predictive value, and negative predictive value. DeLong test was used to compare the AUC values of the same predictive indices between the training cohort and validation cohort to verify the stability of predictive efficacy. Net Reclassification Improvement (NRI) and Integrated Discrimination Improvement (IDI) were employed to evaluate the incremental predictive value of adding PNI and GNRI to the baseline risk model. Additionally, subgroup analyses were conducted to assess the predictive value of PNI and GNRI in different populations, including age (<75 years vs. ≥75 years), gender (male vs. female), ACS type (UA vs. NSTEMI vs. STEMI), diabetes (yes vs. no), and chronic kidney disease (yes vs. no). Statistical analyses were performed using SPSS 26.0 (IBM Corp, Armonk, NY, USA) and R software version 4.3.0. A two-sided *p* < 0.05 was considered statistically significant.

## 3 Results

### 3.1 Baseline characteristics of training cohort

The training cohort included a total of 638 ACS patients, with a mean age of 64.5 ± 11.3 years, and 435 male patients (68.2%). Based on the optimal PNI cutoff value (45.2) and GNRI cutoff value (98.0) determined by maximum Youden’s index of ROC analysis, patients were stratified into low and high nutritional index groups, respectively. Table 1 presents the baseline characteristics of the enrolled patients. Clinically and statistically significant intergroup differences were primarily concentrated in demographic features, comorbidities, cardiac function, inflammatory and nutritional indicators, and coronary lesion severity. Specifically, patients in the low PNI and low GNRI groups were significantly older, had a higher female proportion, and higher burdens of hypertension, diabetes, chronic kidney disease, and previous myocardial infarction (all *p* < 0.05). For cardiac and angiographic characteristics, malnourished patients exhibited lower LVEF, higher syntax scores, and a higher proportion of triple-vessel disease and Killip grade ≥2 (all *p* < 0.001). In terms of laboratory indicators, the low nutritional index groups showed significantly lower serum albumin and lymphocyte counts, accompanied by markedly elevated inflammatory markers (CRP) and heart failure biomarkers (NT-proBNP) (all *p* < 0.001). Notably, only the GNRI grouping showed a significant difference in BMI, with the low GNRI group presenting substantially lower BMI than the high GNRI group (*p* < 0.001). No significant differences in postoperative medication were observed between groups. The overall median hospital stay of the training cohort was 7 (5, 9) days. Subgroup analysis showed that the median hospital stay was numerically longer in low nutritional index groups, but no statistical difference was observed between PNI/GNRI subgroups (*p* = 0.062 and *p* = 0.058, respectively), ensuring the comparability of in-hospital MACE event rates.

**Table 1:** Baseline characteristics of the training cohort grouped by PNI and GNRI.

Characteristics	Total (*n* = 638)	PNI < 45.2 (*n* = 182)	PNI ≥ 45.2 (*n* = 456)	*p*-value	GNRI < 98.0 (*n* = 191)	GNRI ≥ 98.0 (*n* = 447)	*p*-value
Demographic characteristics							
Age, (Years)	64.5 ± 11.3	70.2 ± 10.1	62.2 ± 10.9	<0.001	71.8 ± 9.8	61.5 ± 10.5	<0.001
Gender, (Male)	435 (68.2%)	104 (57.1%)	331 (72.6%)	<0.001	112 (58.6%)	323 (72.3%)	0.001
BMI, (kg/m^2^)	24.6 ± 3.4	23.9 ± 3.8	24.9 ± 3.2	0.002	22.1 ± 3.0	25.7 ± 2.9	<0.001
Comorbidities							
Hypertension, (%)	412 (64.6%)	133 (73.1%)	279 (61.2%)	0.004	139 (72.8%)	273 (61.1%)	0.005
Diabetes, (%)	224 (35.1%)	84 (46.2%)	140 (30.7%)	<0.001	88 (46.1%)	136 (30.4%)	<0.001
Previous myocardial infarction, (%)	76 (11.9%)	32 (17.6%)	44 (9.6%)	0.005	34 (17.8%)	42 (9.4%)	0.003
Chronic kidney disease, (%)	57 (8.9%)	27 (14.8%)	30 (6.6%)	0.001	29 (15.2%)	28 (6.3%)	<0.001
Peripheral arterial disease, (%)	41 (6.4%)	17 (9.3%)	24 (5.3%)	0.061	19 (9.9%)	22 (4.9%)	0.021
Smoke, (%)	291 (45.6%)	71 (39.0%)	220 (48.2%)	0.033	75 (39.3%)	216 (48.3%)	0.036
Clinical presentation							
ACS types				<0.001			<0.001
UA, (%)	354 (55.5%)	84 (46.2%)	270 (59.2%)		90 (47.1%)	264 (59.1%)	
NSTEMI, (%)	143 (22.4%)	42 (23.1%)	101 (22.1%)		44 (23.0%)	99 (22.1%)	
STEMI, (%)	141 (22.1%)	56 (30.8%)	85 (18.6%)		57 (29.8%)	84 (18.8%)	
Killip grade ≥ 2, (%)	117 (18.3%)	53 (29.1%)	64 (14.0%)	<0.001	56 (29.3%)	61 (13.6%)	<0.001
LVEF, (%)	57.2 ± 9.8	54.1 ± 10.7	58.4 ± 9.1	<0.001	54.3 ± 10.5	58.4 ± 9.2	<0.001
Coronary angiographic characteristics							
Triple vessel disease, (%)	162 (25.4%)	61 (33.5%)	101 (22.1%)	0.003	64 (33.5%)	98 (21.9%)	0.002
Syntax score	18.4 ± 9.5	22.1 ± 10.3	17.0 ± 8.8	<0.001	21.8 ± 10.2	17.0 ± 8.9	<0.001
Laboratory examinations							
White blood cell count, (×10^9^/L)	7.8 ± 2.7	8.5 ± 3.1	7.5 ± 2.4	<0.001	8.3 ± 3.0	7.6 ± 2.5	0.002
Neutrophil count, (×10^9^/L)	5.3 ± 2.5	6.5 ± 2.8	4.8 ± 2.2	<0.001	6.2 ± 2.7	4.9 ± 2.2	<0.001
Lymphocyte count, (×10^9^/L)	1.8 ± 0.7	1.3 ± 0.5	2.0 ± 0.6	<0.001	1.5 ± 0.6	1.9 ± 0.7	<0.001
Hemoglobin, (g/L)	135.4 ± 16.8	126.5 ± 17.2	138.9 ± 15.3	<0.001	127.2 ± 16.9	138.9 ± 15.4	<0.001
Serum albumin, (g/L)	38.7 ± 4.6	33.6 ± 3.2	40.8 ± 3.4	<0.001	34.2 ± 3.5	40.7 ± 3.5	<0.001
Creatinine, (μmol/L)	79.2 ± 28.5	88.5 ± 38.1	75.6 ± 22.8	<0.001	87.1 ± 36.8	76.0 ± 23.7	<0.001
CRP, (mg/L)	4.2 (1.8–10.7)	9.5 (4.1–18.3)	3.2 (1.4–7.5)	<0.001	8.9 (3.7–17.5)	3.2 (1.4–7.6)	<0.001
NT-proBNP, (pg/mL)	328 (125–987)	687 (246–1853)	254 (103–653)	<0.001	642 (234–1720)	261 (106–686)	<0.001
Drug therapy							
Aspirin, (%)	627 (98.3%)	179 (98.4%)	448 (98.2%)	0.923	188 (98.4%)	439 (98.2%)	0.849
P_2_Y₁₂ inhibitor, (%)	638 (100%)	182 (100%)	456 (100%)	-	191 (100%)	447 (100%)	-
Beta blockers, (%)	547 (85.7%)	148 (81.3%)	399 (87.5%)	0.045	156 (81.7%)	391 (87.5%)	0.058
ACEI/ARB, (%)	458 (71.8%)	123 (67.6%)	335 (73.5%)	0.140	130 (68.1%)	328 (73.4%)	0.169
Statins, (%)	621 (97.3%)	175 (96.2%)	446 (97.8%)	0.251	184 (96.3%)	437 (97.8%)	0.331
Nutritional assessment indicators							
PNI	47.5 ± 5.6	41.0 ± 3.3	50.2 ± 3.9	<0.001	42.1 ± 4.4	49.8 ± 4.4	<0.001
GNRI	101.6 ± 8.2	93.7 ± 7.1	104.7 ± 6.4	<0.001	92.3 ± 4.7	105.6 ± 6.0	<0.001
Hospitalization stay, (days)	7 (5, 9)	8 (6, 10)	7 (5, 8)	0.062	8 (6, 11)	7 (5, 8)	0.058

Data are presented as mean ± standard deviation, median (interquartile range), or number (percentage). The optimal cutoff values of PNI and GNRI were determined by ROC curve analysis with the maximum Youden’s index (sensitivity+specificity-1) as the screening criterion. BMI, Body Mass Index; UA, Unstable Angina; NSTEMI, Non-ST-segment Elevation Myocardial Infarction; STEMI, ST-segment Elevation Myocardial Infarction; LVEF, Left Ventricular Ejection Fraction; CRP, C-reactive Protein; NT-proBNP, N-terminal pro-Brain Natriuretic Peptide; ACEI, Angiotensin-Converting Enzyme Inhibitor; ARB, Angiotensin II Receptor Blocker; PNI, Prognostic Nutritional Index; GNRI, Geriatric Nutritional Risk Index.

### 3.2 Association between nutritional indicators and in-hospital MACE

**Figure 1:**
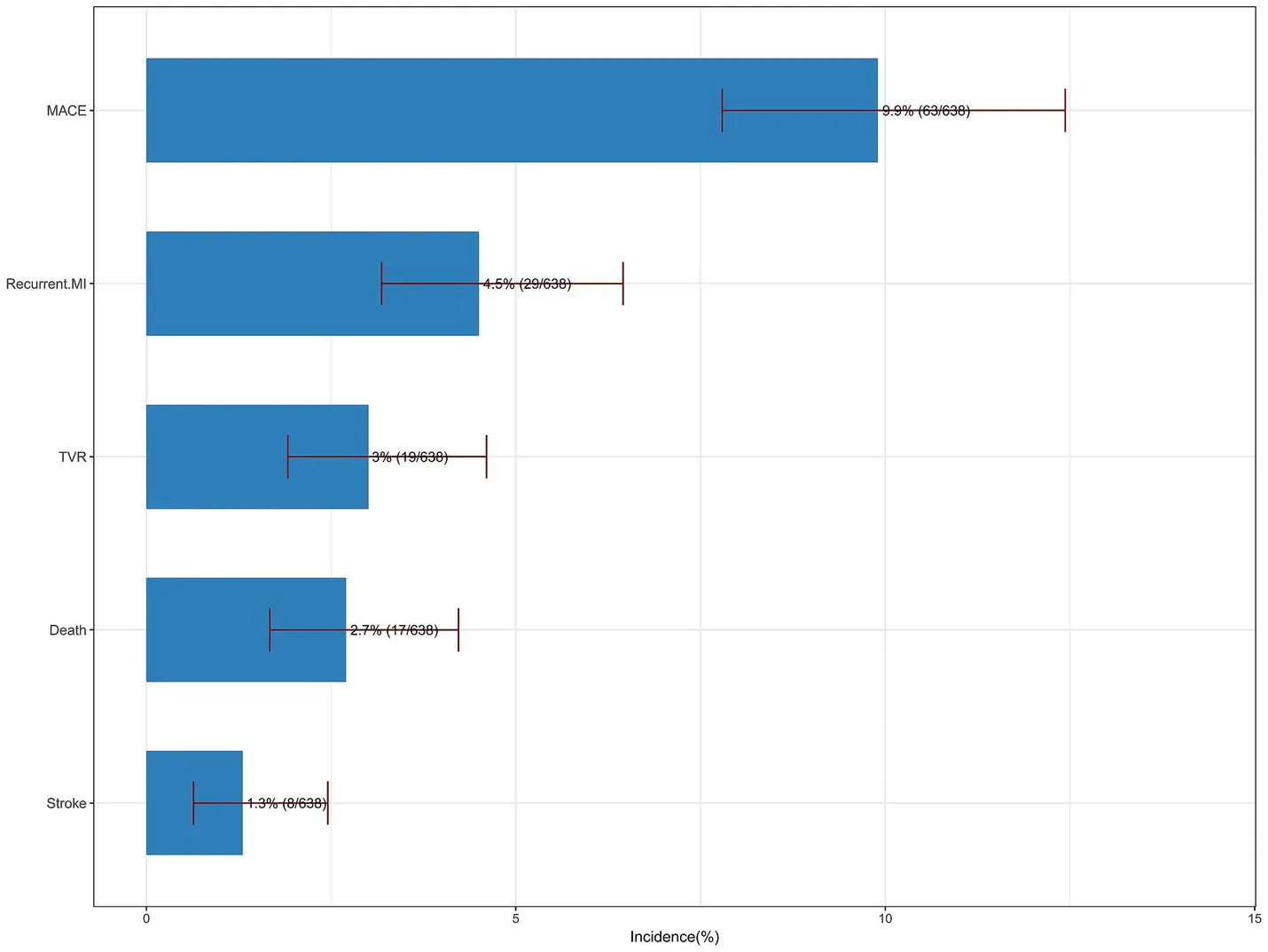
In-hospital MACE and component events in training cohort. MACE, Major Adverse Cardiovascular Events; MI, Myocardial Infarction; TVR, Target vessel revascularization.

**Figure 2:**
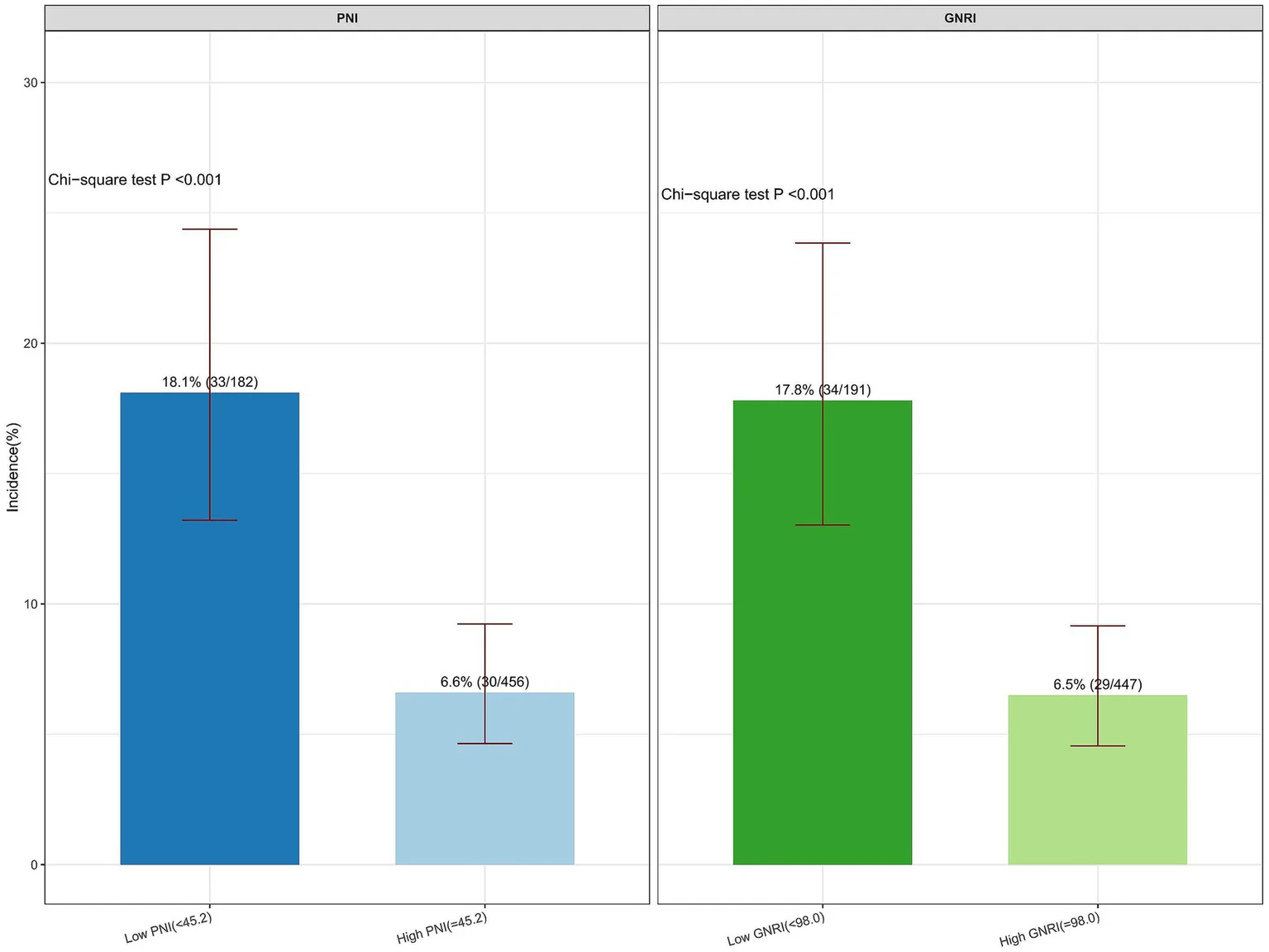
Comparison of the incidence of in-hospital MACE in different nutritional index groups. The optimal cut-off values of PNI (45.2) and GNRI (98.0) were determined by ROC curve analysis based on the maximum Youden’s index in the training cohort. PNI, Prognostic Nutritional Index; GNRI, Geriatric Nutritional Risk Index.

Among the 638 patients in the training cohort, 63 (9.9%) experienced in-hospital MACE, including 17 cases of all-cause mortality (2.7%), 29 cases of recurrent myocardial infarction (4.5%), 8 cases of stroke (1.3%), and 19 cases of target vessel revascularization (3.0%) (Figure 1). The incidence of in-hospital MACE in the low PNI group was significantly higher than that in the high PNI group (18.1% vs. 6.6%, *p* < 0.001). Similarly, the incidence of in-hospital MACE in the low GNRI group was also significantly higher than that in the high GNRI group (17.8% vs. 6.5%, *p* < 0.001) (Figure 2). Multivariate logistic regression analysis showed that after adjusting for potential confounding factors, both low PNI (<45.2) and low GNRI (<98.0) were independent predictors of in-hospital MACE (Table 2). Low PNI was associated with a 2.37-fold increase in the risk of in-hospital MACE (adjusted OR = 2.37, 95%CI: 1.42–3.96, *p* = 0.001), while low GNRI was associated with a 2.18-fold increase in the risk of in-hospital MACE (adjusted OR = 2.18, 95%CI: 1.31–3.64, *p* = 0.003). In addition, comprehensive VIF multicollinearity analysis was performed for all confounding variables included in the multivariate regression model. All covariates exhibited VIF values below 3.0 (far less than the diagnostic threshold of 5.0), among which PNI (VIF = 2.745) and GNRI (VIF = 2.618) showed relatively higher values due to their shared albumin component, yet no significant multicollinearity was detected. This further validates the reliability of the elevated OR values of PNI and GNRI in the adjusted models and excludes statistical interference from collinearity (Supplementary Table 1).

**Table 2:** Multivariate logistic regression analysis: association of PNI and GNRI with in-hospital MACE (training cohort).

Variables	Univariate analysis	*p*-value	Multivariate analysis*	*p*-value
	OR (95% CI)		OR (95% CI)	
PNI				
PNI ≥ 45.2	References		References	
PNI < 45.2	3.14 (1.94–5.09)	<0.001	2.37 (1.42–3.96)	0.001
GNRI				
GNRI≥98.0	References		References	
GNRI<98.0	3.13 (1.93–5.07)	<0.001	2.18 (1.31–3.64)	0.003

*Adjusted factors: age, sex, BMI, ACS type, Killip classification, hypertension, diabetes, previous myocardial infarction, chronic kidney disease, LVEF, three-vessel disease, Syntax score, CRP and NT-proBNP. The cutoff values of PNI and GNRI were determined by the maximum Youden’s index based on training cohort ROC analysis. OR, Odds ratio; CI, Confidence interval; PNI, Prognostic Nutritional Index; GNRI, Geriatric Nutritional Risk Index; MACE, Major Adverse Cardiovascular Events.

### 3.3 Comparison of the ability of nutritional indicators to predict in-hospital MACE

**Figure 3:**
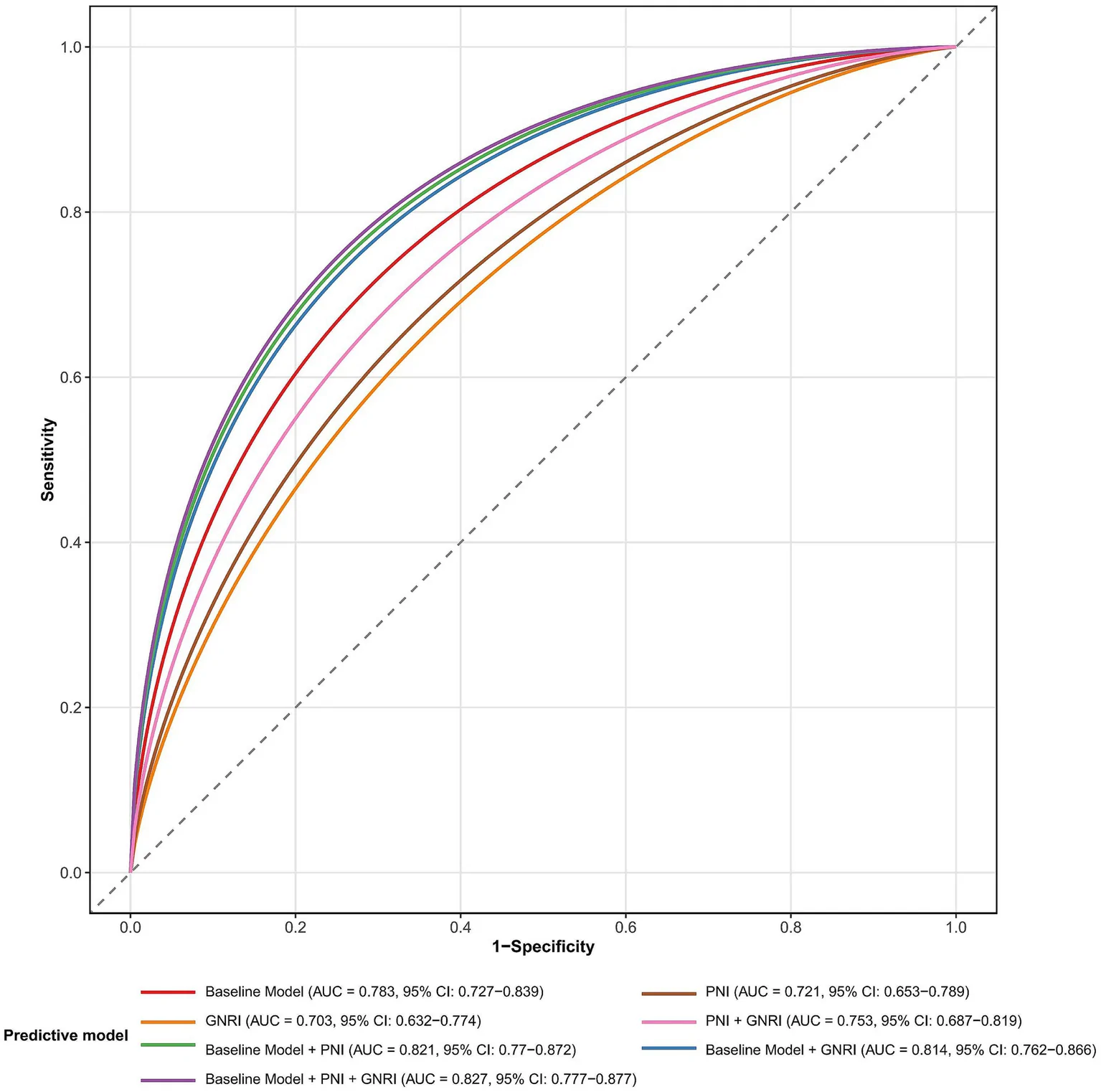
Receiver operating characteristic (ROC) curves for prediction of in-hospital major adverse cardiovascular events. The x-axis represents 1-specificity and the y-axis represents sensitivity. Different colored lines represent each predictive model with their respective area under the curve (AUC) values and 95% confidence intervals. The optimal cut-off values of PNI (45.2) and GNRI (98.0) for predicting in-hospital MACE were determined by ROC curve analysis with the maximum Youden’s index in the training cohort. The baseline model includes: Age, sex, ACS type, Killip classification, hypertension, diabetes, previous myocardial infarction, chronic kidney disease, LVEF, three-vessel disease and syntax score. PNI, Prognostic Nutritional Index; GNRI, Geriatric Nutritional Risk Index.

ROC curve analysis showed that both PNI and GNRI had good predictive ability for in-hospital MACE (Figure 3). The AUC for PNI was 0.721 (95%CI: 0.653–0.789), and for GNRI was 0.703 (95%CI: 0.632–0.774), with no significant difference between the two (*p* = 0.247). The optimal cutoff value for PNI was 45.2, with a sensitivity of 57.1% and specificity of 76.3%; the optimal cutoff value for GNRI was 98.0, with a sensitivity of 55.6% and specificity of 73.9%. The baseline model based on clinical risk factors (including age, gender, ACS type, Killip classification, hypertension, diabetes, previous myocardial infarction, chronic kidney disease, LVEF, three-vessel disease, and syntax score) had an AUC of 0.783 (95%CI: 0.727–0.839). Adding either PNI or GNRI to the baseline model significantly improved predictive ability: the AUC for baseline model + PNI was 0.821 (95%CI: 0.770–0.872, *p* = 0.008 vs. baseline model); the AUC for baseline model + GNRI was 0.814 (95%CI: 0.762–0.866, *p* = 0.014 vs. baseline model). The combination of both nutritional indices (PNI + GNRI) had an AUC of 0.753 (95%CI: 0.687–0.819), which was higher than either index alone but did not reach statistical significance. The AUC for adding both indices simultaneously to the baseline model was 0.827 (95%CI: 0.777–0.877), which was significantly higher than the baseline model (*p* = 0.004).

To further quantify the incremental clinical value of nutritional indicators beyond traditional AUC evaluation, Net Reclassification Improvement (NRI) and Integrated Discrimination Improvement (IDI) were calculated. Clinically, NRI reflects the accuracy improvement of individual patient risk reclassification after adding new predictive indicators, while IDI represents the overall enhancement of the model’s ability to distinguish patients with and without adverse events. These two indicators can effectively verify the true clinical net benefit of model optimization, compensating for the limitation that AUC only reflects overall discriminatory efficacy. NRI and IDI analyses also confirmed the incremental value of adding nutritional indices to the baseline model. The NRI for adding PNI was 0.283 (*p* = 0.002) and the IDI was 0.037 (*p* = 0.003); the NRI for adding GNRI was 0.246 (*p* = 0.007) and the IDI was 0.031 (*p* = 0.006); the NRI for adding both indices simultaneously was 0.318 (*p* < 0.001) and the IDI was 0.045 (*p* < 0.001).

### 3.4 Subgroup analyses

**Figure 4:**
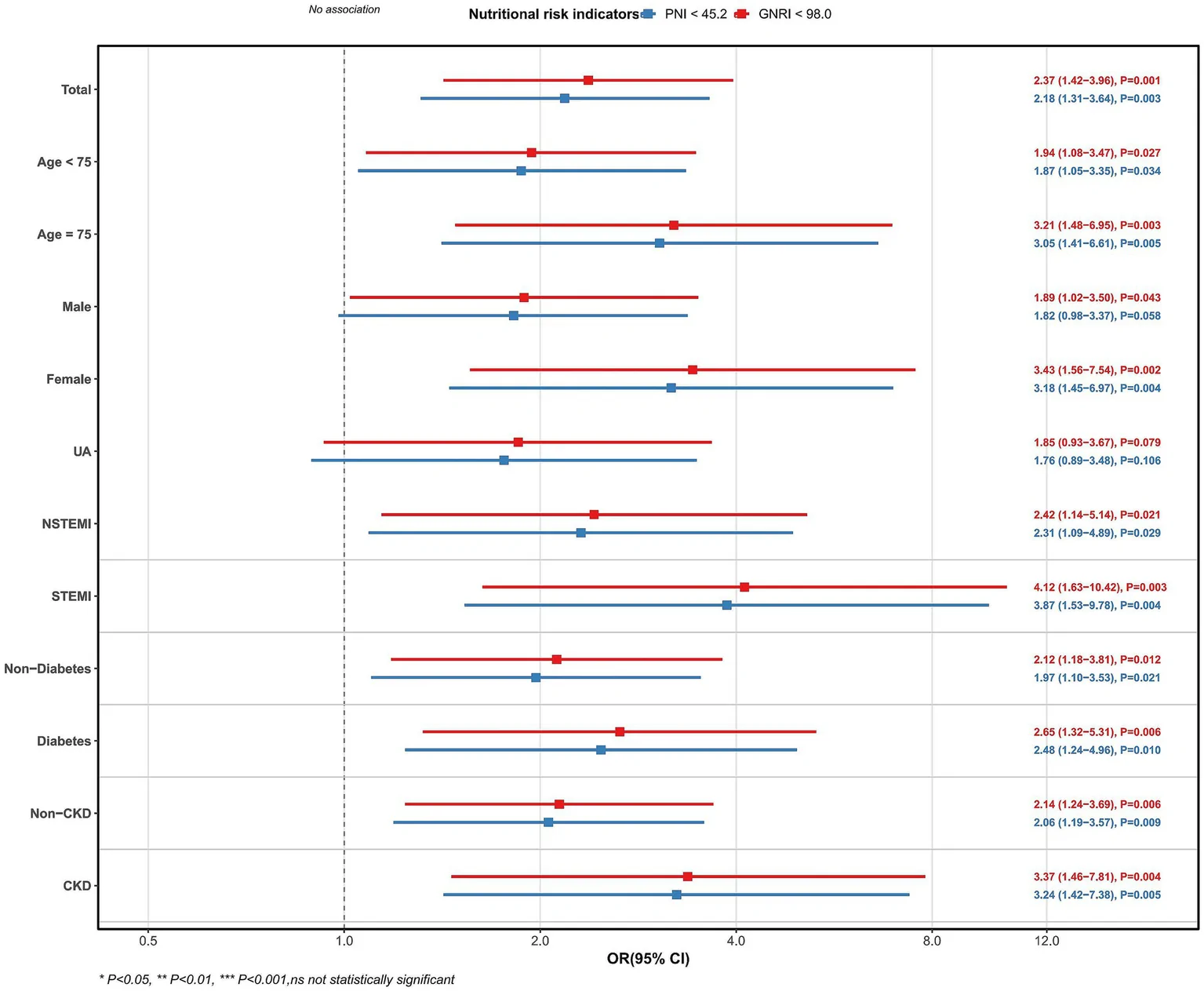
Subgroup analysis of the association between nutritional indices and in-hospital major adverse cardiovascular events. For each subgroup, the forest plot displays separate odds ratios for PNI (blue squares) and GNRI (red circles) with corresponding 95% confidence intervals (horizontal lines). *p*-values for interaction between subgroups are shown in the rightmost column. Patients were stratified into low and high PNI/GNRI groups according to ROC-derived optimal cut-off values (PNI = 45.2, GNRI = 98.0) based on maximum Youden’s index in the training cohort. PNI, Prognostic Nutritional Index; GNRI, Geriatric Nutritional Risk Index; UA, Unstable Angina; NSTEMI, Non-ST-segment Elevation Myocardial Infarction; STEMI, ST-segment Elevation Myocardial Infarction; CKD, Chronic kidney disease.

Subgroup analyses were conducted to evaluate the predictive value of PNI and GNRI in different populations (Figure 4). The results showed that the predictive value of PNI and GNRI was more significant in elderly patients (≥75 years), females, and STEMI patients. In elderly patients, low PNI (adjusted OR = 3.21, 95%CI: 1.48–6.95, *p* = 0.003) and low GNRI (adjusted OR = 3.05, 95%CI: 1.41–6.61, *p* = 0.005) were both associated with a higher risk of in-hospital MACE. In female patients, the predictive value of low PNI (adjusted OR = 3.43, 95%CI: 1.56–7.54, *p* = 0.002) and low GNRI (adjusted OR = 3.18, 95%CI: 1.45–6.97, *p* = 0.004) was also prominent. In STEMI patients, the association between nutritional deficiency indicators and in-hospital MACE was even stronger (low PNI: adjusted OR = 4.12, 95%CI: 1.63–10.42, *p* = 0.003; low GNRI: adjusted OR = 3.87, 95%CI: 1.53–9.78, *p* = 0.004) (Table 3).

**Table 3:** Comparison of different prediction models (training cohort).

Predictive models	AUC (95% CI)	*p*-value*	Sensitivity	Specificity	PPV	NPV	NRI	*p*-value	IDI	*p*-value
Baseline model	0.783 (0.727–0.839)	-	71.4%	73.2%	22.7%	95.9%	-	-	-	-
PNI	0.721 (0.653–0.789)	0.035	57.1%	76.3%	20.8%	94.2%	-	-	-	-
GNRI	0.703 (0.632–0.774)	0.019	55.6%	73.9%	18.8%	93.8%	-	-	-	-
PNI + GNRI	0.753 (0.687–0.819)	0.247^	65.1%	76.0%	22.5%	95.3%	-	-	-	-
Baseline model + PNI	0.821 (0.770–0.872)	0.008	77.8%	75.3%	25.5%	97.0%	0.283	0.002	0.037	0.003
Baseline model + GNRI	0.814 (0.762–0.866)	0.014	74.6%	76.9%	26.1%	96.5%	0.246	0.007	0.031	0.006
Baseline model + PNI + GNRI	0.827 (0.777–0.877)	0.004	79.4%	76.5%	27.0%	97.2%	0.318	<0.001	0.045	<0.001

*p value compared with the baseline model; ^*p* value compared with PNI. DeLong test was used for AUC comparison between different models. The baseline model includes: age, sex, ACS type, Killip classification, hypertension, diabetes, previous myocardial infarction, chronic kidney disease, LVEF, three-vessel disease and syntax score. AUC, Area Under Curve; CI, Confidence interval; PPV, Positive Predictive Value; NPV, Negative Predictive Value; NRI, Net Reclassification Improvement; IDI, Integrated Discrimination Improvement; PNI, Prognostic Nutritional Index; GNRI, Geriatric Nutritional Risk Index.

### 3.5 Validation cohort results

To verify the reliability of the association between PNI, GNRI and in-hospital MACE, 450 ACS patients (validation cohort) from Hefei Second People’s Hospital and Ma’anshan People’s Hospital were analyzed. First, we compared the baseline characteristics of patients from the two sub-centers in the validation cohort (Supplementary Table 2). There were no significant differences in age, gender, BMI, comorbidities, laboratory indicators, coronary lesion characteristics and medication status between the two groups (all *p* > 0.05), which confirmed the rationality of pooling the two hospital populations for subsequent analysis. The baseline characteristics of the overall validation cohort were balanced and consistent with the training cohort, with good population representativeness. In the validation cohort, 42 patients (9.3%) experienced in-hospital MACE. The median hospital stay of the validation cohort was 7 (5, 8) days, with no significant difference between low and high PNI/GNRI subgroups (all *p* > 0.05) (Supplementary Table 3). Table 4 shows the association of PNI and GNRI with in-hospital MACE in the validation cohort. Consistent with the results of the training cohort, multivariate analysis showed that low PNI (adjusted OR = 2.25, 95%CI: 1.19–4.27, *p* = 0.013) and low GNRI (adjusted OR = 2.09, 95%CI: 1.10–3.97, *p* = 0.024) were both independent predictors of in-hospital MACE in the validation cohort. The ROC curve analysis results were also similar to the training cohort, with an AUC of 0.708 (95%CI: 0.623–0.793) for PNI and an AUC of 0.694 (95%CI: 0.608–0.780) for GNRI, with no significant difference between the two (*p* = 0.382). DeLong test results showed that there was no significant difference in AUC values of PNI (*p* = 0.352) and GNRI (*p* = 0.379) between the training cohort and validation cohort. The slight numerical decrease of AUC in the validation cohort was normal statistical fluctuation, and the predictive efficacy of the two nutritional indices remained stable and consistent across different populations.

**Table 4:** Multivariate logistic regression analysis: association of PNI and GNRI with in-hospital MACE (validation cohort).

Variables	Univariate analysis	*p*-value	Multivariate analysis*	*p*-value
	OR (95% CI)		OR (95% CI)	
PNI				
PNI ≥ 45.2	References		References	
PNI < 45.2	3.02 (1.63–5.58)	<0.001	2.25 (1.19–4.27)	0.013
GNRI				
GNRI≥98.0	References		References	
GNRI<98.0	2.95 (1.60–5.43)	0.001	2.09 (1.10–3.97)	0.024

*The baseline characteristics of the two sub-centers in the validation cohort (Hefei Second People’s Hospital, *n* = 212; Ma’anshan People’s Hospital, *n* = 238) were balanced (all *p* > 0.05), and the pooled population was representative. DeLong test for AUC comparison between training and validation cohorts: PNI: *p* = 0.352; GNRI: *p* = 0.379. The median hospital stay of the validation cohort was 7 (5, 8) days, with no significant difference between low and high PNI/GNRI subgroups (all *p* > 0.05). Adjusted factors: age, sex, BMI, ACS type, Killip classification, hypertension, diabetes, previous myocardial infarction, chronic kidney disease, LVEF, three-vessel disease, syntax score, CRP and NT-proBNP. OR, Odds ratio; CI, Confidence interval; PNI, Prognostic Nutritional Index; GNRI, Geriatric Nutritional Risk Index; MACE, Major Adverse Cardiovascular Events.

## 4 Discussion

This study is the first to systematically evaluate the association between PNI, GNRI and in-hospital MACE events in Chinese multicenter ACS populations undergoing PCI treatment. The study found that low PNI (<45.2) and low GNRI (<98.0) were both independent predictors of in-hospital MACE, a result that was confirmed in the validation cohort. The two nutritional indicators had similar predictive abilities, and incorporating them into traditional clinical risk models significantly improved the predictive value for in-hospital MACE. Additionally, these nutritional indicators demonstrated stronger predictive value in high-risk populations such as the elderly, females, and STEMI patients.

Notably, the present study further clarifies the fundamental mechanistic differences and complementary clinical values between PNI and GNRI, which distinguishes our findings from previous single-index or mechanistically undifferentiated studies. Most prior ACS-related studies only focused on the individual prognostic efficacy of PNI or GNRI, without distinguishing their unique physiological implications and complementary predictive advantages in acute cardiovascular events (2, 5, 9). In essence, PNI and GNRI integrate overlapping but distinct clinical parameters, enabling them to reflect two mutually interactive but independent pathological dimensions in ACS patients.

PNI, calculated based on serum albumin and peripheral lymphocyte count, is a typical nutrition-inflammation composite index that preferentially reflects short-term acute inflammatory stress and real-time immune function status during acute ACS hospitalization (24). Combined with hypoalbuminemia caused by acute inflammatory consumption, reduced PNI dynamically captures the severity of in-hospital acute inflammatory response, which is closely correlated with short-term in-hospital adverse cardiovascular events. In contrast, GNRI incorporates serum albumin and anthropometric parameters (actual/ideal body weight ratio), which is insensitive to acute transient inflammatory fluctuations and mainly reflects long-term baseline nutritional reserve, somatic energy storage, and chronic physical tolerance of patients (7, 25). Weight-derived indicators in GNRI can stably evaluate chronic malnutrition and lean mass loss that accumulate over a long period, which cannot be fully reflected by acute hematological indexes such as lymphocyte count.

This complementary mechanism is the core reason why the combined application of PNI and GNRI achieves superior predictive performance than single index in our study. Previous studies mostly ignored this synergistic predictive logic: PNI captures short-term acute inflammatory immune disturbance after ACS onset, while GNRI evaluates long-term chronic nutritional reserve deficiency before disease onset. The combination of the two indices can simultaneously cover “chronic baseline vulnerability + acute in-hospital stress injury”, forming a more comprehensive risk evaluation system for ACS patients undergoing PCI, which fills the gap of insufficient differentiated evaluation of nutritional-inflammatory status in previous domestic and foreign studies.

The results of this study are generally consistent with previous literature reports. Studies have found that PNI < 41.1 is an independent predictor of in-hospital death in STEMI patients (OR = 3.11), similar to our finding that the low PNI group had a 2.37-fold increased risk of MACE. Chen et al., in a study including 5,006 coronary heart disease patients undergoing PCI, found that the low PNI (<45.5) group had significantly higher long-term all-cause mortality (HR = 2.45) (26). Regarding GNRI, Shibata et al. showed that GNRI<92 is an independent predictor of 30-day mortality in patients undergoing transcatheter aortic valve replacement (12). Notably, the optimal cutoff value for GNRI in our study (98.0) is higher than some previous studies, which may reflect differences in optimal cutoff values across different disease states and population characteristics (27). Compared with previous single-center studies with limited sample size and single-index evaluation, our multicenter validation data further confirms that although the two indices have similar overall predictive AUC values for in-hospital MACE, their internal predictive mechanisms are heterogeneous and complementary, which provides a novel theoretical basis for dual nutritional-inflammatory risk stratification in ACS patients. Malnutrition may affect the prognosis of ACS patients through multiple mechanisms. First, malnutrition status is closely associated with systemic inflammation. In our study, patients in both the low PNI and low GNRI groups had significantly elevated hs-CRP levels, supporting this view. Second, hypoalbuminemia may affect the prognosis of ACS patients by weakening vascular endothelial function, increasing thrombotic tendency, and influencing drug distribution and effects (28, 29). Third, malnourished patients often have decreased immune function, which may increase the risk of postoperative infections and other complications (6). Additionally, lymphocytopenia may reflect immunosuppression under stress conditions and is associated with increased risk of cardiovascular events (30).

This study found that the predictive value of PNI and GNRI was more significant in elderly patients, females, and STEMI patients, which has important clinical implications. This subgroup difference further verifies the complementary mechanism of the two indices: elderly and female patients are more prone to chronic nutritional reserve insufficiency (abnormal GNRI) and have weaker immune stress regulation ability, while STEMI patients suffer from more severe acute inflammatory stress response (abnormal PNI), resulting in significantly amplified predictive efficacy of dual nutritional-inflammatory indicators in these high-risk populations. Elderly patients often have baseline malnutrition or risk of malnutrition, with studies showing that over 60% of elderly ACS patients have varying degrees of nutritional problems (31). Female ACS patients have different clinical presentations and outcomes compared to males, which may be related to gender differences in baseline metabolism and inflammatory responses (32). STEMI patients face more severe ischemia–reperfusion injury and stress responses, which may make the impact of nutritional status on prognosis more prominent (33). These findings suggest that clinicians should pay special attention to nutritional assessment and intervention in these high-risk populations.

Incorporating PNI and GNRI into clinical risk assessment models can significantly improve the predictive value for in-hospital MACE, a finding consistent with Kang et al.’s research on nutritional scores enhancing the predictive ability of traditional risk assessment tools (34). Our study shows that although adding either indicator alone to the baseline model significantly improves predictive ability, adding both indicators simultaneously achieves the best predictive effect, suggesting they may provide complementary information. Indeed, PNI focuses on reflecting acute immune-inflammatory stress status (short-term in-hospital pathological change), while GNRI pays more attention to chronic overall nutritional reserve status (long-term baseline physical condition); combining the two can avoid the one-sidedness of single index evaluation and provide a comprehensive assessment of the superposition effect of chronic malnutrition and acute inflammation stress in ACS patients. Combining the two may provide a more comprehensive assessment of nutritional status.

The results of this study have important clinical implications. First, both PNI and GNRI are simple and readily available indicators that can be quickly calculated based on routine examinations without additional costs and special equipment. Second, identifying patients at high risk for malnutrition helps clinicians implement targeted interventions. For clinical practice, targeted intervention strategies can be formulated based on the index abnormalities: for patients with low PNI, anti-inflammatory and immune regulatory adjuvant therapy can be strengthened to improve acute stress status; for patients with low GNRI, early nutritional support can be implemented to supplement long-term nutritional reserves, which provides precise individualized intervention ideas for ACS postoperative management. Third, these indicators can assist clinical decision-making; for example, high-risk patients with low PNI and low GNRI may require more aggressive drug therapy and closer monitoring. This study has several limitations. First, as a retrospective study, it cannot rule out the influence of unmeasured confounding factors. Second, this study only evaluated in-hospital MACE events and did not provide long-term follow-up data. Third, although validation was conducted across three hospitals, all were from Anhui Province, China, which may limit the generalizability of the results. Fourth, the study was unable to obtain detailed nutritional intake and body composition data, factors that may affect the accuracy of nutritional status assessment.

To overcome the limitations of the current retrospective study and validate its clinical application value, future research should design large-sample, multicenter prospective studies with long-term follow-up. Further prospective exploration of dynamic nutritional index changes will help establish a more accurate, standardized, and generalizable nutritional risk stratification and rehabilitation management system for ACS patients. In conclusion, this multicenter retrospective study verifies that PNI and GNRI are independent and complementary predictors of in-hospital MACE in PCI-treated ACS patients. PNI reflects acute inflammatory-immune stress, while GNRI reflects chronic nutritional reserve, and their combination significantly improves the predictive efficacy of traditional risk models, particularly in elderly, female, and STEMI high-risk populations. With excellent accessibility and clinical operability, the two indicators possess great translational potential for standardized perioperative nutritional screening, individualized intervention, and postoperative rehabilitation optimization.

## Data Availability

The original contributions presented in the study are included in the article/Supplementary material, further inquiries can be directed to the corresponding authors.
