# Supplementary material for: Prognostic nutritional index and geriatric nutritional risk index as predictors of in-hospital major adverse cardiovascular events after percutaneous coronary intervention in acute coronary syndrome patients: a multicenter retrospective cohort study
Source: Front Nutr. 2026 Aug 26;13:1848510. doi: 10.3389/fnut.2026.1848510 (PMC13553417; doi:10.3389/fnut.2026.1848510)
Supplement: Supplementary file 1 [file Table_1.doc]

**Supplementary Table 1. Multicollinearity Test of All Variables from Table 1 (Variance Inflation Factor, VIF)**

| **Variables** | **VIF Value** | **Tolerance** |
| --- | --- | --- |
| **Demographic characteristics** | | |
| Age | 1.624 | 0.616 |
| Gender (Male) | 1.287 | 0.777 |
| BMI | 1.352 | 0.74 |
| **Comorbidities** | | |
| Hypertension | 1.198 | 0.835 |
| Diabetes | 1.245 | 0.803 |
| Previous myocardial infarction | 1.172 | 0.853 |
| Chronic kidney disease | 1.384 | 0.723 |
| Peripheral arterial disease | 1.156 | 0.865 |
| Smoke | 1.134 | 0.882 |
| **Clinical presentation** | | |
| ACS type | 1.419 | 0.705 |
| Killip grade ≥2 | 1.536 | 0.651 |
| LVEF | 1.467 | 0.682 |
| **Coronary angiographic characteristics** | | |
| Triple vessel disease | 1.329 | 0.752 |
| Syntax score | 1.508 | 0.663 |
| Laboratory examinations |  |  |
| White blood cell count | 2.147 | 0.466 |
| Neutrophil count | 2.315 | 0.432 |
| Lymphocyte count | 1.982 | 0.504 |
| Hemoglobin | 1.426 | 0.701 |
| Serum albumin | 2.473 | 0.404 |
| Creatinine | 1.361 | 0.735 |
| CRP | 1.573 | 0.636 |
| NT-proBNP | 1.691 | 0.591 |
| **Drug therapy** | | |
| Aspirin | 1.042 | 0.96 |
| P2Y₁₂ inhibitor | 1 | 1 |
| Beta blockers | 1.128 | 0.887 |
| ACEI/ARB | 1.095 | 0.913 |
| Statins | 1.076 | 0.929 |
| **Nutritional assessment indicators** | | |
| PNI | 2.745 | 0.364 |
| GNRI | 2.618 | 0.382 |
| Hospitalization stay |  |  |
| Hospitalization stay (days) | 1.237 | 0.808 |

VIF: Variance Inflation Factor; Tolerance = 1/VIF. The table presents multicollinearity test results for all variables listed in Table 1. All VIF values were below 3.0 (far less than the conventional diagnostic threshold of 5.0), indicating no significant multicollinearity among variables. Notably, PNI and GNRI showed relatively higher VIF values (2.745 and 2.618, respectively) due to shared serum albumin parameter, but both remained well within the statistically acceptable range. Among laboratory parameters, white blood cell count and neutrophil count showed mild collinearity as expected, which did not affect model stability.

**Supplementary Table 2.Baseline characteristics of the two sub-centers in the validation cohort**

| **Characteristics** | **Hefei Second People's Hospital (n=212)** | **Ma'anshan People's Hospital (n=238)** | **P-Value** |
| --- | --- | --- | --- |
| **Demographic characteristics** | | | |
| Age,(Years) | 63.8±10.9 | 64.2±11.1 | 0.702 |
| Gender,(Male) | 145 (68.4%) | 162 (68.1%) | 0.942 |
| BMI,(kg/m²) | 24.5±3.3 | 24.7±3.5 | 0.531 |
| Comorbidities |  |  |  |
| Hypertension,(%) | 137 (64.6%) | 154 (64.7%) | 0.982 |
| Diabetes,(%) | 74 (34.9%) | 83 (34.9%) | 0.997 |
| Previous myocardial infarction,(%) | 25 (11.8%) | 28 (11.8%) | 0.991 |
| Chronic kidney disease,(%) | 19 (9.0%) | 21 (8.8%) | 0.952 |
| **Clinical presentation** | | | |
| ACS Types |  |  | 0.976 |
| UA,(%) | 118 (55.7%) | 132 (55.5%) |  |
| NSTEMI,(%) | 47 (22.2%) | 53 (22.3%) |  |
| STEMI,(%) | 47 (22.2%) | 53 (22.3%) |  |
| Killip grade≥2,(%) | 39 (18.4%) | 43 (18.1%) | 0.932 |
| LVEF,(%) | 57.4±9.7 | 57.1±9.9 | 0.741 |
| **Laboratory examinations** | | | |
| Serum albumin,(g/L) | 38.5±4.5 | 38.6±4.7 | 0.803 |
| Lymphocyte count,(×10⁹/L) | 1.8±0.7 | 1.8±0.6 | 0.872 |
| CRP,(mg/L) | 4.3 (1.7-10.9) | 4.2 (1.8-10.5) | 0.891 |
| Hospitalization stay, (days) | 7 (5, 8) | 7 (5, 8) | 0.763 |
| **Nutritional assessment indicators** | | | |
| PNI | 47.3±5.5 | 47.4±5.7 | 0.842 |
| GNRI | 101.4±8.1 | 101.5±8.3 | 0.895 |

Data are presented as mean ± standard deviation, median (interquartile range), or number (percentage). BMI:Body Mass Index; UA:Unstable Angina; NSTEMI:Non-ST-segment Elevation Myocardial Infarction; STEMI:ST-segment Elevation Myocardial Infarction; LVEF:Left Ventricular Ejection Fraction; CRP:C-reactive Protein; PNI:Prognostic Nutritional Index; GNRI:Geriatric Nutritional Risk Index.

**Supplementary Table 3.Baseline characteristics of the overall validation cohort grouped by PNI and GNRI**

| **Characteristics** | **Total(n=450)** | **PNI<45.2 (n=126)** | **PNI≥45.2 (n=324)** | **P-Value** | **GNRI<98.0 (n=134)** | **GNRI≥98.0 (n=316)** | **P-Value** |
| --- | --- | --- | --- | --- | --- | --- | --- |
| **Demographic characteristics** | | | | | | | |
| Age,(Years) | 64.0±11.0 | 69.8±10.2 | 61.7±10.8 | <0.001 | 71.5±9.9 | 61.3±10.4 | <0.001 |
| Gender,(Male) | 307 (68.2%) | 72 (57.1%) | 235 (72.5%) | 0.002 | 79 (59.0%) | 228 (72.1%) | 0.004 |
| BMI,(kg/m²) | 24.6±3.4 | 23.8±3.7 | 24.9±3.2 | 0.003 | 22.0±3.0 | 25.7±2.9 | <0.001 |
| **Comorbidities** | | | | | | | |
| Hypertension,(%) | 291 (64.7%) | 92 (73.0%) | 199 (61.4%) | 0.013 | 97 (72.4%) | 194 (61.4%) | 0.015 |
| Diabetes,(%) | 157 (34.9%) | 58 (46.0%) | 99 (30.6%) | 0.001 | 61 (45.5%) | 96 (30.4%) | 0.001 |
| Previous myocardial infarction,(%) | 53 (11.8%) | 22 (17.5%) | 31 (9.6%) | 0.013 | 24 (17.9%) | 29 (9.2%) | 0.005 |
| Chronic kidney disease,(%) | 40 (8.9%) | 19 (15.1%) | 21 (6.5%) | 0.003 | 20 (14.9%) | 20 (6.3%) | 0.002 |
| **Clinical presentation** | | | | | | | |
| ACS Types |  |  |  | <0.001 |  |  | <0.001 |
| UA,(%) | 250 (55.6%) | 58 (46.0%) | 192 (59.3%) |  | 63 (47.0%) | 187 (59.2%) |  |
| NSTEMI,(%) | 100 (22.2%) | 29 (23.0%) | 71 (21.9%) |  | 31 (23.1%) | 69 (21.8%) |  |
| STEMI,(%) | 100 (22.2%) | 39 (31.0%) | 61 (18.8%) |  | 40 (29.9%) | 60 (19.0%) |  |
| Killip grade≥2,(%) | 82 (18.2%) | 37 (29.4%) | 45 (13.9%) | <0.001 | 39 (29.1%) | 43 (13.6%) | <0.001 |
| LVEF,(%) | 57.2±9.8 | 54.0±10.6 | 58.5±9.0 | <0.001 | 54.2±10.4 | 58.5±9.1 | <0.001 |
| Hospitalization stay, (days) | 7 (5, 8) | 8 (6, 10) | 7 (5, 8) | 0.071 | 8 (6, 10) | 7 (5, 8) | 0.065 |
| **Nutritional assessment indicators** | | | | | | | |
| PNI | 47.4±5.6 | 40.9±3.2 | 50.2±3.9 | <0.001 | 42.0±4.3 | 49.8±4.4 | <0.001 |
| GNRI | 101.5±8.2 | 93.6±7.0 | 104.7±6.4 | <0.001 | 92.2±4.6 | 105.6±6.0 | <0.001 |

Data are presented as mean ± standard deviation, median (interquartile range), or number (percentage). The optimal cutoff values of PNI and GNRI were determined by the maximum Youden’s index based on training cohort ROC analysis. BMI:Body Mass Index; UA:Unstable Angina; NSTEMI:Non-ST-segment Elevation Myocardial Infarction; STEMI:ST-segment Elevation Myocardial Infarction; LVEF:Left Ventricular Ejection Fraction; PNI:Prognostic Nutritional Index; GNRI:Geriatric Nutritional Risk Index.
